# Neuropsychiatric events in SLE: determination of attribution and assessment of outcome

**DOI:** 10.1186/ar3952

**Published:** 2012-09-27

**Authors:** JG Hanly

**Affiliations:** 1 Capital Health and Dalhousie University, Halifax, NS, Canada

## Background

Neuropsychiatric (NP) events are common in SLE patients and in the general population. Although some events are a direct result of lupus-related autoimmune and inflammatory mechanisms, many are not. The frequency and diversity of NP events in SLE patients pose a challenge in determining their correct attribution and assessing outcome. These are of fundamental importance for the care of individual patients, studies of pathogenesis and the conduct of clinical trials.

## Methods

A total of 1,826 patients have been enrolled within 15 months of SLE diagnosis into an international, long-term prospective cohort study. Patients are assessed annually for up to 10 years. At each assessment, all NP events as per the ACR case definitions of 19 NP syndromes are recorded. Attribution of each NP event is determined centrally using two composite attribution rules of different stringency. Additional data collection includes demographic and clinical variables, SLE global disease activity (SLEDAI-2K), SLICC/ACR damage index (SDI), physician assessment of NP events (Likert scale) and self-report mental (MCS) and physical (PCS) component summary scores of the SF-36. Statistical analyses include Cox proportional hazards model, Kaplan-Meier curves, regression and multi-state statistical models.

## Results

Upon enrollment into the cohort the proportion of NP events attributed to SLE varied from 19 to 38% and affected 6.1 to 11.7% of patients, depending upon the stringency of the attribution rule. Seizure disorders, cerebrovascular disease, acute confusional states and neuropathies were the most common of the 19 NP syndromes. Changes in SF-36 summary and subscale scores, in particular those related to mental health, were strongly associated with physician assessment of clinical outcomes of NP events. The short-term outcome of NP events was determined by their characteristics and attribution: focal events and those attributed to SLE had better outcomes. Overall, NP events had a negative impact on self-report health-related quality of life (HRQoL) but this was not necessarily true for all types of NP events. For example, most seizures were attributed to SLE, resolved in the absence of anti-seizure medications and were not associated with a persistent negative impact on HRQoL.

## Conclusion

The clinical and epidemiological challenge imposed by the high frequency and diversity of NP events in SLE patients can be addressed through the application of composite attribution rules, as well as physician and patient-derived outcome measures.

